# Long non-coding RNA FAM133B-2 represses the radio-resistance of nasopharyngeal cancer cells by targeting miR-34a-5p/CDK6 axis

**DOI:** 10.18632/aging.103600

**Published:** 2020-09-05

**Authors:** Dabing Huang, Xianhai Zhu, Yong Wang, Haobin Yu, Youguang Pu

**Affiliations:** 1 Department of Oncology, The First Affiliated Hospital of USTC, Division of Life Sciences and Medicine, University of Science and Technology of China, Hefei 230001, Anhui Province, P.R. China; 2 Department of Interventional Oncology, Anhui Provincial Cancer Hospital, West Branch of the First Affiliated Hospital of USTC, Division of Life Sciences and Medicine, University of Science and Technology of China, Hefei 230001, Anhui Province, P.R. China; 3 Department of Cancer Nutrition and Metabolic Therapy, No.3 Ward of Oncology, Anhui Provincial Cancer Hospital, West Branch of the First Affiliated Hospital of USTC, Division of Life Sciences and Medicine, University of Science and Technology of China, Hefei 230001, Anhui Province, P.R. China; 4 Department of Cancer Epigenetics Program, Anhui Provincial Cancer Hospital, West Branch of the First Affiliated Hospital of USTC, Division of Life Sciences and Medicine, University of Science and Technology of China, Hefei 230001, Anhui Province, P.R. China

**Keywords:** nasopharyngeal carcinoma, radio-resistance, lncRNA FAM133B-2, miR-34a-5p, CDK6

## Abstract

Long non-coding RNAs (lncRNAs) were found to play roles in various cancers, including nasopharyngeal carcinoma. In this study, we focused on the biological function of the lncRNA FAM133B-2 in the radio-resistance of nasopharyngeal carcinoma. The RNA-seq and qRT-PCR analysis showed that FAM133B-2 is highly expressed in the radio-resistant nasopharyngeal carcinoma cells. The following biochemical assays showed that FAM133B-2 represses the nasopharyngeal carcinoma radio-resistance and also affects the apoptosis and proliferation of nasopharyngeal carcinoma cells. Further investigations suggested that miR-34a-5p targets FAM133B-2 and also regulates the cyclin-dependent kinase 6 (CDK6). All these results suggested that the lncRNA FAM133B-2 might function as a competitive endogenous RNA (ceRNA) for miR-34a-5p in nasopharyngeal carcinoma radio-resistance, thus it may be regarded as a novel prognostic biomarker and therapeutic target in nasopharyngeal carcinoma diagnosis and treatment.

## INTRODUCTION

Nasopharyngeal carcinoma (NPC), which commonly occurs in the epithelial lining of the nasopharynx, is one of the most common types of head and neck tumors [1, 2]. Currently, radio/chemo-therapy and radiotherapy are the primary methods for the treatment of NPC, but they are less efficient due to the high sensitivity of NPC [3]. Moreover, despite extensive studies on the radio-resistance of cancers, the molecular mechanism for NPC radio-resistance remains largely unknown. Hence, further investigations are needed to elucidate the molecular mechanism of NPC radio-resistance and more valid therapeutic strategies are authoritatively required.

Long non-coding RNAs (lncRNAs) are a group of RNA transcripts of more than 200 nucleotides that generally do not encode proteins [4]. Evidence has suggested that lncRNAs have diverse functions, such as the regulators of transcription; modulators of mRNA processing, post-transcriptional control; and organization of nuclear domains [5, 6]. Moreover, given their sophisticated nature, lncRNAs have been implicated in the development of diseases. Specifically, in cancer, numerous studies have shown that many lncRNAs are closely associated with the development of various cancers [7–10]. To date, several lncRNAs, including EWSAT1 [11], LOC100129148 [12], PCAT7 [13], CCAT1 [14], NCK1-AS1 [15], and LINC00460 [16] are found to involve in NPC tumorigenesis, and some of them have been identified as alternative therapeutic targets and biomarkers for NPC. Notably, the lncRNA THOR was found to attenuate cisplatin sensitivity of NPC cells [17]. However, the fine molecular mechanism of lncRNA-regulated radio-resistance of NPC remains unclear.

In this study, we identified a new lncRNA, termed FAM133B-2, which was significantly upregulated in the radio-resistant NPC cells. We also found that the forced reversal of FAM133B-2 level is closely related to the NPC radio-resistance. Moreover, our data revealed that FAM133B-2 is a target of miR-34a-5p, which in return negatively regulates the expression of CDK6 gene. All these results suggested that the lncRNA FAM133B-2 might function as a competitive endogenous RNA (ceRNA) for miR-34a-5p in NPC radio-resistance, thus it may represent a feasible biomarker for diagnosis and treatment of NPC radio-resistance.

## RESULTS

### Identification and characteristics of radio-resistant NPC cells of CNE-2R and 6-10BR

**Figure 1:**
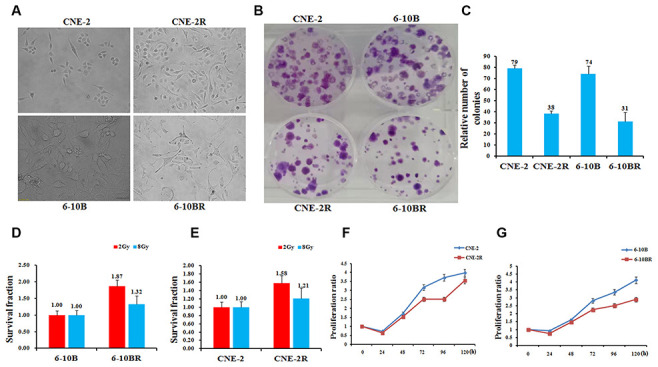
**Establishment, identification and biological characteristics of radiotherapy resistant strains of nasopharyngeal cancer cells.** (**A**) Cell morphology identification. CNE-2R and 6-10BR cell lines were established from CNE-2 and 6-10B, respectively. The cumulative dose of radiation of CNE-2R and 6-10BR reached to 80 and 76Gy, respectively. Under the optical microscope, the morphology of the cell line was obviously changed. (**B** and **C**) CNE-2R, 6-10BR and their parental cells were subjected to a sphere formation assay. The sphere numbers were determined after seven days for the first generation (G1) and seven days after seeding for G2. Treatment with SCF was repeated when the cells were passaged. The data are mean±SD of two independent experiments. (**D** and **E**) Radiosensitivity detection assay showed that the sensitivity of CNE-2R and 6-10BR cells was lower than that of parental cells. (**F** and **G**) Cell proliferation assay showed that the proliferation of CNE-2R and 6-10BR cells was slower than that of parental cells.

To set up a platform for investigating the mechanism of NPC radio-resistance, we first screened the radio-resistant NPC cells by radio treatment. The parental cells CNE-2 and 6-10B were subjected to X-ray radiation at increasing doses. After several rounds of screening against X-ray challenge, we successfully obtained two mutated NPC strains that are radio-resistant compared to the parental strains. They can tolerate the X-ray radiation at a dose up to 80 and 76Gy, respectively. We thus termed them as CNE-2R and 6-10BR respectively. During the X-ray challenge, the morphology of the cell lines was obviously altered (Figure 1A). Compared to the parental cells, the shape of CNE-2R cells became irregular, whereas the size of 6-10BR cells were much smaller due to shrinking.

We then compared the features of these two new cell lines to their parental cells by a series of biochemical assays. The sphere formation assays showed that the CNE-2R and 6-10BR cells possess only half number of the colonies compared to their parental cells (Figure 1B and 1C). Next, we detected the radio-sensitivity of CNE-2R and 6-10BR cells. Upon radio treatment at 2Gy and 8Gy, respectively, both CNE-2R and 6-10BR cells showed an increased survival fraction, compared to their parental cells (Figure 1D and 1E). The results showed that CNE-2R and 6-10BR cells indeed confer the capability of radio-resistance, and thus has a lower sensitivity against radio treatment. Moreover, the cell proliferation assays showed that CNE-2R and 6-10BR cells have a lower proliferation rate compared to their parental cells (Figure 1F and 1G). All these results clearly demonstrated that the radio-resistant cells CNE-2R and 6-10BR showed a substantial difference compared to the parental cells.

### FAM133B-2 represses the radio-resistance of NPC cells

**Figure 2:**
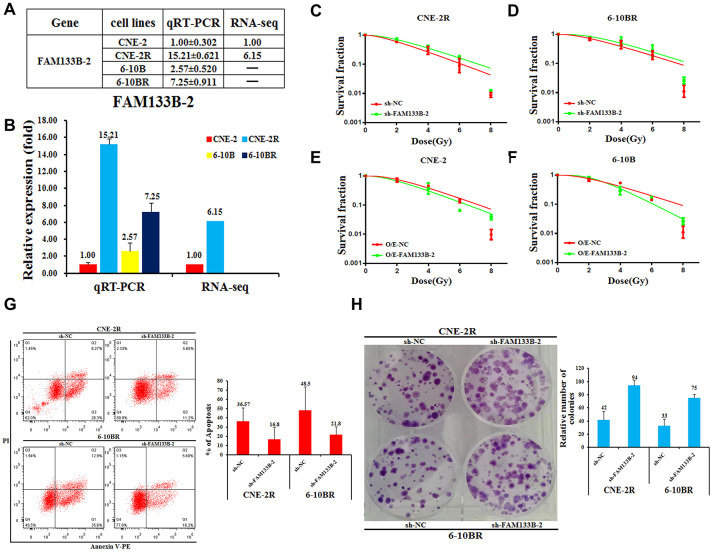
**Effects of a forced reversal of FAM133B-2 level on the nasopharyngeal cancer cells.** The relative FAM133B-2 level (fold) in CNE-2R and 6-10BR cells versus CNE-2 and 6-10B cells measured by both miR-omic and qRT-PCR analyses is shown in a table (**A**) and those measured by qRT-PCR are shown in a plot (**B**). “-” indicates no detection in the omic analysis. sh-FAM133B-2-transfected CNE-2R (**C**) and 6-10BR (**D**) cells survival fraction versus the negative control (NC) cells for 24h, then cells were digested and counted according to 500 (0Gy), 1000 (2Gy), 2000 (4Gy), 5000 (6Gy), 8000 (8Gy) cells/well and was inoculated in a 6-well plate in triplicate, the corresponding dose was irradiated after 24h, using a 6-MV x-ray generated by a linear accelerator Varian trilogy at a dose rate of 2Gy/min (Varian trilogy at a dose rate of 2Gy/min). CNE-2 (**E**) and 6-10B (**F**) cells infected with FAM133B-2-O/E versus the negative control (NC-O/E), then were digested and counted according to 500 (0Gy), 1000 (2Gy), 2000 (4Gy), 5000 (6Gy), 8000 (8Gy) cells/well and was inoculated in a 6-well plate in triplicate, the corresponding dose was irradiated after 24h, using a 6-MV x-ray generated by a linear accelerator Varian trilogy at a dose rate of 2Gy/min (Varian trilogy at a dose rate of 2Gy/min). (**G**) The effects of the forced reversal of FAM133B-2 level on the apoptosis of CNE-2R and 6-10BR cells by FACS analysis in plot and in the original with a graph of the analyzed data and plots of the original FACS data. (**H**) The effects of the forced reversal of FAM133B-2 level on the sphere numbers of CNE-2Rand 6-10BR cells. The sphere numbers were determined after seven days for the first generation (G1) and seven days after seeding for G2. Treatment with SCF was repeated when the cells were passaged. Colony formation numbers, relative sphere formation are shown. The sphere formation assays showed that the sphere numbers of CNE-2R and 6-10BR cells was fewer than that of parental cells. The data are mean±SD of two independent experiments. The surviving fraction was calculated using the multitarget single-hit model: Y=1-(1-exp(-k*x))^N. The data are presented as the mean±standard deviation of results from 3 independent experiments, and two way Anova was used to calculate statistical significance.

To find the molecular insights that involve in the radio-resistance of NPC cells, we performed the lncRNA-seq analysis of radio-sensitive CNE-2 and radio-resistant CNE-2R cells, and compared the differentially expressed genes. The results gave several lncRNAs that differ at least 2-folds of the expression in the two cells. Among them, FAM133B-2 is one of the most significantly differentially expressed genes, which has a 6-fold higher expression in CNE-2R compared to CNE-2 cells (Figure 2A and 2B Supplementary Figures 1, 3). Moreover, the qRT-PCR analysis showed that the expression of FAM133B-2 is much higher in the radio-resistant CNE-2R and 6-10BR cells, which are 15.21- and 2.82-folds compared to CNE-2 and 6-10B, respectively (Figure 2A and 2B).

Next, we reversely changed the FAM133B-2 level in the NPC cell lines to check the effect on NPC radio-resistance. First, we down-regulated the FAM133B-2 level in either CNE-2R or 6-10BR cells by transfecting sh-FAM133B-2 in the cells. Accompanied by the decrease of FAM133B-2 level in either CNE-2R or 6-10BR cells, the cell survival rate was slightly increased upon the radio treatment at 2, 4, 6, and 8Gy (Figure 2C and 2D). In contrast, we up-regulated the FAM133B-2 level in either CNE-2 or 6-10B cells by over-expressing FAM133B-2 in the cells. The results showed a lower survival rate upon the up-regulation of FAM133B-2 in either CNE-2 or 6-10B cells (Figure 2E and 2F). The results clearly demonstrated that FAM133B-2 represses the radio-resistance of NPC cells.

To further investigate the effects of a forced reversal of FAM133B-2 in CNE-2R and 6-10BR cells, we detected the apoptosis rate by FACS analysis. Upon the decrease of FAM133B-2 in either CNE-2R or 6-10BR cells, the number of apoptotic cells significantly decreased, with the apoptosis rate decreased from 36.57% to 16.80% in CNE-2R cells, and from 48.50% to 21.80% in 6-10BR cells (Figure 2G). Furthermore, the colony formation assays revealed that down-regulation of FAM133B-2 in CNE-2R and 6-10BR cells in return significantly increased the number of colonies to over 2-folds (Figure 2H). The results are also in agreement with the notion that a lower level of FAM133B-2 promotes the radio-resistance of NPC cells, which has a much higher proliferation rate, as shown by the colony formation assays.

### FAM133B-2 is a target of miR-34a-5p in NPC cells

**Figure 3:**
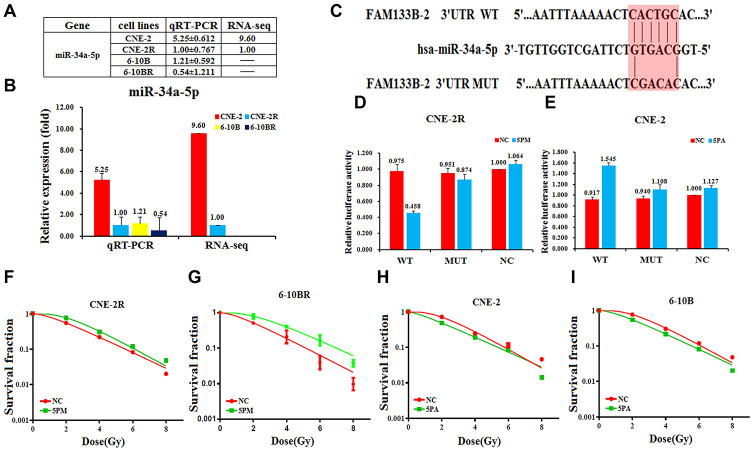
**FAM133B-2 is a target of miR-34a-5p in nasopharyngeal cancer cells.** The relative miR-34a-5p level (fold) in CNE-2R and 6-10BR cells versus CNE-2 and 6-10B cells measured by both miR-omic and qRT-PCR analyses is shown in a table (**A**) and those measured by qRT-PCR are shown in a plot (**B**). “-” indicates no detection in the omic analysis. (**C**) Luciferase reporter constructs: WT and MUT FAM133B-2 in the miR-34a-5p-binding sites were inserted into psiCHECK-2 vector. The red region is the binding site. The FAM133B-2 site is predicted to be a target of miR-34a-5p. One seed sequence mutant of miR-34a-5p was shown below. (**D** and **E**)The relative luciferase activities (fold) of the reporter with the wild-type (WT) or mutant-type (MUT) FAM133B-2-UTR or without the UTR (NC) were determined in the nasopharyngeal cancer cells transfected with the miR-34a-5p mimic (in CNE-2R) or antagomiR (in CNE-2). (**F** and **G**) MiR-34a-5p mimic (5PM)-transfected CNE-2R and 6-10BR increases survival fraction versus the negative control (NC) cells. (**H** and **I**) MiR-34a-5p antagomiR (5PA)-transfected CNE-2 and 6-10B decreases NC cells survival fraction versus the negative control (NC) cells.

Previous reports suggested that lncRNAs could act as competing endogenous RNAs (ceRNAs) to sponge miRNAs and thus regulate cancer progression [18, 19]. We proposed that FAM133B-2 might be a ceRNA to sponge miRNA. Moreover, we previously found that the miRNA miR-34a-5p involves in the radio- or drug-resistance of cancers [20, 21]. To test whether miR-34a-5p involves in the NPC radio-resistance, we first tested the expression of miR-34a-5p in the NPC cells by RNA-seq and qRT-PCR analysis Supplementary Figure 3. The results showed that the miR-34a-5p level is much higher in the radio-sensitive CNE-2 and 6-10B cells, compared to the radio-resistant CNE-2R and 6-10BR cells (Figure 3A and 3B). The expression of miR-34a-5p is negatively correlated with the FAM133B-2 level in the NPC cells, indicating FAM133B-2 might be a target of miR-34a-5p. Sequence analysis showed that the 3’-UTR region of FAM133B-2 has a sequence motif that is complementary with miR-34a-5p (Figure 3C). We thus performed the luciferase reporter assays by constructing the wild-type or miR-34a-5p binding-site mutant of FAM133B-2 in the plasmid. We then tested the luciferase activity by transfecting the miR-34a-5p mimic in the CNE-2R cells or the miR-34a-5p antagomiR in the CNE-2 cells. Upon the up-regulation of miR-34a-5p in the CNE-2R cells, the luciferase activity was reduced to about half of the level in the wild-type cells, indicating a reduced expression of FAM133B-2 (Figure 3D). However, mutation of the miR-34a-5p binding site in FAM133B-2 almost abolished the reducing effect, which showed a comparable luciferase activity (Figure 3D). In contrast, down-regulation of miR-34a-5p in the CNE-2 cells increased the expression of FAM133B-2 to 1.5 folds compared to the control. As expected, mutation of the miR-34a-5p binding site in FAM133B-2 also has a minor effect on the FAM133B-2 expression in CNE-2 cells (Figure 3E). All these results suggested that FAM133B-2 is a target of miR-34a-5p in NPC cells, which negatively regulates the expression of FAM133B-2.

Next, we tested whether the reversal change of miR-34a-5p level may affect the radio-resistance of NPC cells. We tested the radio-resistance capability by transfecting the miR-34a-5p mimic in either the CNE-2R or 6-10BR cells, or miR-34a-5p antagomiR in either CNE-2 or 6-10B cells. Accompanied by the increase of miR-34a-5p in the CNE-2R or 6-10BR cells, the cell survival rate largely increased, indicating a lower sensitivity against the radio treatment (Figure 3F and 3G). In contrast, the cell survival rate is somewhat decreased upon down-regulation of miR-34a-5p in either CNE-2 or 6-10B cells (Figure 3H and 3I). The results also negatively correlate with the effect of a forced change of FAM133B-2 in NPC cells. Accordingly, miR-34a-5p negatively regulates the FAM133B-2 level in NPC cells, which might be a ceRNA to sponge miR-34a-5p function on NPC radio-resistance.

### The cyclin-dependent kinase 6 is a target of miR-34a-5p in NPC cells

**Figure 4:**
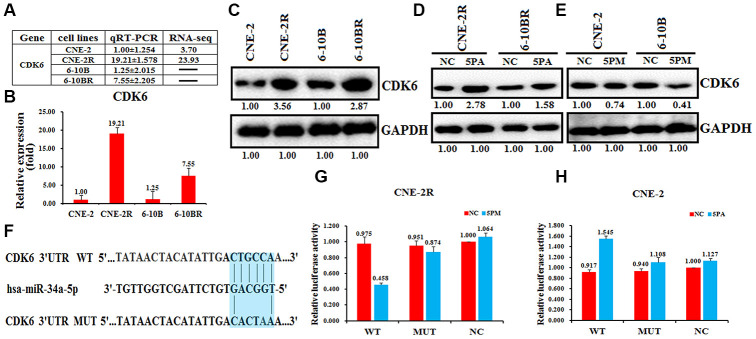
**CDK6 is a target of miR-34a-5p in nasopharyngeal cancer cells.** The relative CDK6 level (fold) in CNE-2R and 6-10BR cells versus CNE-2 and 6-10B cells measured by miR-omic shown in a table (**A**), qRT-PCR shown in a plot (**B**) and western analyses shown in (**C**). “-” indicates no detection in the omic analysis. CDK6 protein levels determined western blot analyses in the miR-34a-5p antagomiR (5PA) -transfected CNE-2R and 6-10BR cells (**D**) and the miR-34a-5p mimic (5PM)-transfected CNE-2 and 6-10B cells (**E**) versus the negative control (NC). (**F**) Luciferase reporter constructs: WT and MUT CDK6 in the miR-34a-5p-binding sites were inserted into psiCHECK-2 vector. The blue region is the binding site. The CDK6 site is predicted to be a target of miR-34a-5p. One seed sequence mutant of miR-34a-5p was shown below. The relative luciferase activities (fold) of the reporter with the wild-type (WT) or mutant-type (MUT) CDK6-UTR or without the UTR (NC) were determined in the nasopharyngeal cancer cells transfected with the miR-34a-5p mimic (in CNE-2R) (**G**) or antagomiR (in CNE-2) (**H**).

The cyclin-dependent kinase 6 (CDK6) was found to involve in the drug resistance [22–24]. Moreover, we found that CDK6 mRNA level is higher in the radio-resistant CNE-2R and 6-10BR cells, compared to the radio-sensitive CNE-2 or 6-10B cells (Figure 4A and 4B Supplementary Figure 3). The western blot analysis also showed a higher protein level in CNE-2R or 6-10BR cells (Figure 4C). The CDK6 level is negatively correlated with the miR-34a-5p level, indicating CDK6 might be a target of miR-34a-5p. To further validate this hypothesis, we tested the CDK6 protein expression by transfecting the miR-34a-5p antagomiR in the CNE-2R and 6-10BR cells, or the miR-34a-5p mimic in the CNE-2 and 6-10B cells. As a result, miR-34a-5p knockdown increased the CDK6 protein level by 2.78- and 1.58-folds in CNE-2R and 6-10BR cells, respectively (Figure 4D). Moreover, up-regulation of miR-34a-5p decreased the CDK6 protein level to 74% and 41% in CNE-2 and 6-10B cells, respectively (Figure 4E).

Sequence analysis showed that the 3’-UTR regions of CDK6 and miR-34a-5p share a complementary sequence (Figure 4F). To further test whether CDK6 is a target of miR-34a-5p in NPC cells, we performed the luciferase reporter assays by constructing the wild-type or miR-34a-5p binding-site mutated CDK6 into the psiCHECK-2 vector. As a result, the wild-type construct, but not the mutant showed a much lower luciferase activity upon the increase of miR-34a-5p in CNE-2R cells, compared to the control (Figure 4G). In contrast, miR-34a-5p knockdown in CNE-2 cells increased the luciferase activity of the wild-type construct, but not the mutant (Figure 4F). These results demonstrated that CDK6 is a target of miR-34a-5p in NPC cells.

### FAM133B-2 represses the NPC radio-resistance via regulating miR-34a-5p/CDK6 axis

**Figure 5:**
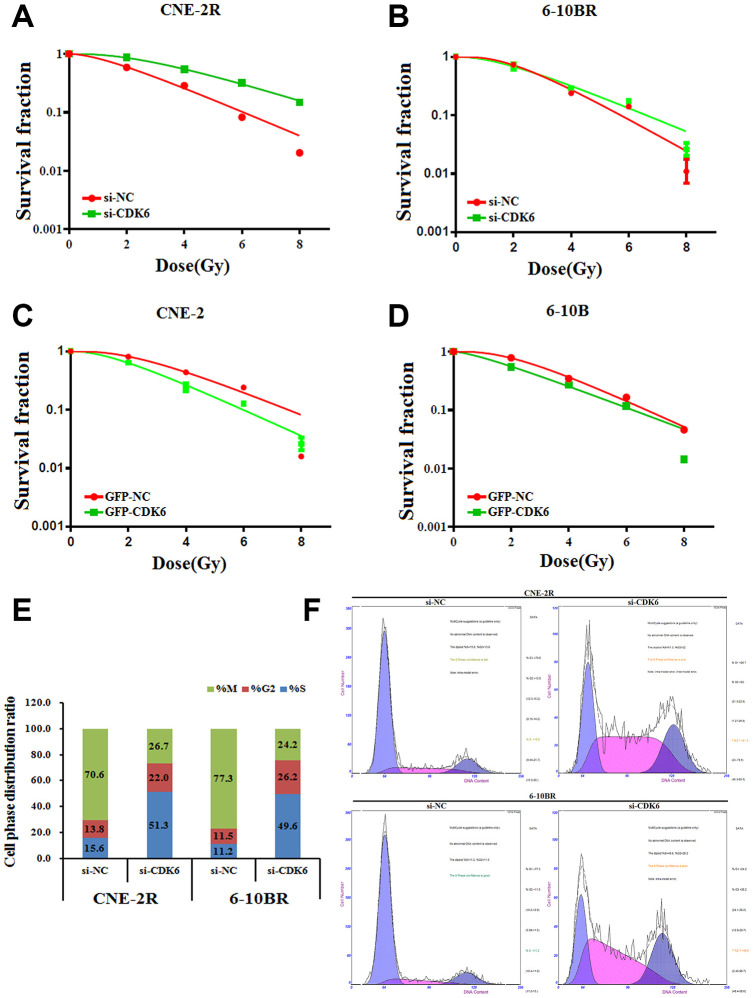
(**A** and **B**) si-CDK6-transfected CNE-2R and 6-10BR increases survival fraction versus the negative control (NC) cells. (**C** and **D**) GFP-CDK6-transfected CNE-2 and 6-10B decreases NC cells survival fraction versus the negative control (NC) cells. The effects of the forced reversal of CDK6 level on the cell cycle distribution of CNE-2R and 6-10BR cells by FACS analysis in plot and in the original (**E** and **F**).

As CDK6 is a target of miR-34a-5p, we thus tested whether CDK6 is the functional target that involves in the NPC radio-resistance. Similarly, we performed the apoptosis assays by the reversal changes of CDK6 level in NPC cells. The results showed that CDK6 knock-down in CNE-2R and 6-10BR cells significantly increased the cell survival rate, indicating a lower sensitivity against radio radiation (Figure 5A and 5B). Moreover, up-regulation of CDK6 in CNE-2 and 6-10B cells decreased the cell survival rate (Figure 5C and 5D). The results confirmed the inhibition effect of CDK6 on NPC radio-resistance, similar to the effect of FAM133B-2. These data also indicated that miR-34a-5p facilitates the NPC radio-resistance via targeting CDK6 and FAM133B-2.

Furthermore, we analyzed the cell cycle distribution by CDK6 knockdown in CNE-2R and 6-10BR cells. Most of the control cells are in the mitotic period, whereas CDK knockdown triggers half of the cells synchronized in the S phase (Figure 5E and 5F). The results indicated that CDK6 affects the cell cycle, which eventually might associate with the NPC radio-resistance.

## DISCUSSION

Increasing studies have reported that lncRNAs play integral and crucial roles in a variety of biological processes [25]. Additionally, accumulating evidences revealed that a dozen of lncRNAs involve in tumorigenesis via regulating many target genes [26–29]. Several lncRNAs were also found to be involved in the NPC tumorigenesis. For example, MALAT1 was found to be upregulated and to modulate the activity of cancer stem cells and radio-resistance by regulating the miR-1/slug axis in NPC [30]. HOTAIR knockdown was shown to repress cell proliferation and invasion in NPC cells, providing an available therapeutic agent for NPC [31, 32]. However, the roles and mechanisms of lncRNAs in NPC radio-resistance remain primarily unknown. Here, we showed that the level of FAM133B-2 was higher in the radio-resistant NPC cells than corresponding parental cells. We also confirmed that silencing of FAM133B-2 repressed NPC cell apoptosis but facilitated NPC cell proliferation. Mechanistically, we showed that the 3’-UTR of FAM133B-2 directly interacts with miR-34a-5p. This is reminiscent of the competing endogenous RNA (ceRNA) hypothesis that lncRNA might function as a molecular sponge of miRNAs to regulate target gene expression [33]. We propose that FAM133B-2 exerts its inhibition function on NPC radio-resistance probably via functioning as a ceRNA for miR-34a-5p, and subsequently initiating CDK6 signaling pathway.

It has been reported that miR-34a-5p also involves in the tumorigenesis of various cancers [20, 34, 35]. Moreover, miR-34a-5p was also found to be associated with lncRNAs to exert their functions. For example, miR-34a-5p expression was reduced by the lncRNA XIST, which functions as an oncogene in NPC [36]. The lncRNA NEAT1 targets miR-34a-5p at least partially to drive NPC progression via Wnt/β-Catenin Signaling [37]. Notably, the lncRNA NEAT1 promotes docetaxel resistance in prostate cancer by regulating ACSL4 via sponging miR-34a-5p and miR-204-5p [34]. Our results together with previous findings supported the ceRNA mechanism for miRNA and lncRNA co-regulated tumorigenesis.

The cyclin-dependent kinase -4 and -6 (CDK4/6) are serine/threonine kinases bound to Cyclin D1, that function as master integrators of G1-S transition of the cell cycle [38–40]. Aberrant regulation of cell cycle is a hallmark of cancer [41, 42]. The CDK4/6 activity is deregulated through various genetic alterations in many human tumors. In agreement with previous findings, our results showed that miR-34a-5p-regulated CDK6 represses the NPC radio-resistance. Moreover, CDK6 knockdown mostly arrested the CNE-2R and 6-10BR cells in the S phase, further confirming the role of CDK6 in cell cycle transition.

In conclusion, our study demonstrated that FAM133B-2 is up-regulated in the radio-resistant NPC cells. Furthermore, we found that FAM133B-2 is targeted by miR-34a-5p, which also negatively regulates the CDK6 expression. All these results provide new insights into NPC radio-resistance, and thus FAM133B-2 might be a potential prognostic and diagnostic marker for NPC treatment.

## MATERIALS AND METHODS

### Cells and culture

The human nasopharyngeal carcinoma cells lines CNE-2 and 6-10B were kindly provided by the Cancer Center of Sun Yat-sen University. CNE-2R and 6-10BR were obtained from their parental strains of CNE-2 and 6-10B, respectively. Four cells were cultured and maintained in RPMI medium 1640 (BI) supplemented with 10% fetal bovine serum (PAN), 1% glutamine (WISENT), 100U/ml penicillin (WISENT), and 100mg/ml streptomycin (WISENT) in humidified air at 37°C with 5% CO_2_.

### Radiation exposure and clonogenic assays

All cells were pretreated by NC, miR-34a-5p mimic, antagomiR, sh-FAM133B-2, FAM133B-2-O/E and si-CDK6 for 24h, then were digested and counted according to 500 (0Gy), 1000 (2Gy), 2000 (4Gy), 5000 (6Gy), 8000 (8Gy) cells/well and was inoculated in a 6-well plate in triplicate, the corresponding dose was irradiated after 24h, using a 6-MV x-ray generated by a linear accelerator (Varian trilogy at a dose rate of 2Gy/min). And the 1640 medium was continued for 15 days, then washed and fixed with 10% formaldehyde, and stained with Giemsa. Only clones with more than 50 cells can be used as the cloned spheres. The number of cloned spheres with>50 cells was counted, and the number of cells inoculated with 50 to 200 cloned spheres was selected as the appropriate number of colonies for colony formation experiments, all experiment repeated 3 times and taken the mean. Calculate the cell clone formation rate and cell survival fraction, using the multi-target click model of GraphPad Prism 6 software.

### Transient transfection assays

The *Homo sapiens* miR-34a-5p mimic, antagomiR, sh-FAM133B-2, si-CDK6 and corresponding scrambled negative control (NC) were obtained from Guangzhou Ribobio, China. All the transfection experiments were performed using riboFECT CP transfection kit were supplied by Guangzhou Ribobio, China. qRT-PCR and western blot assays were performed to confirm the effect of transfection.

### Lentivirus production and infection

HEK293T cells, lentivirus packaging cells or comparable cells were examined and plated so that the cells are 70-80% confluent at the moment of transfection. 2.5μg of lentiviral expression plasmid and 5.0μl of Lenti-Pac HIV were mixed into 200μl of Opti-MEM® I (Invitrogen). 15μl of EndoFectin Lenti was diluted into 200μl of Opti-MEM I. The diluted EndoFectin Lenti reagent was added drop wise to the DNA solution while gently vortexing the DNA-containing tube. The mixture was then incubated for 15min at room temperature to allow DNA-EndoFectin complexes to form. The DNA-EndoFectin Lenti complexes were added directly to each dish, which was gently swirled to distribute the complexes.

### Cell proliferation assay

The capacity for cellular proliferation was measured by CCK8-based cell proliferation assay. Cells were seeded in 96-well plates at a density of 5x10^3^ cells per well, and cell proliferation assays were performed every 24h using CCK8. The number of viable cells was measured by their absorbance at 450nm at the indicated time points.

### Transient transfection assays and reagents

siRNA and scrambled (negative control, NC) sequences as well as a riboFECT CP transfection kit were supplied by Guangzhou RiboBio, China. Transfections of the above mentioned ribonucleic acid reagents were performed according to the manufacturer’s instructions.

### RNA analysis

Total RNA was isolated from the cultured cells with the Trizol (Tiangen). For mRNA analysis, a cDNA primed by an oligo-dT was constructed using HiScript® RII 1^st^ Strand cDNA Synthesis Kit (Vazyme). The RNA level was quantified using duplex-qRT-PCR analysis, Either U6 small nuclear RNA (HmiRQP9001) or β-actin (ShingGene) was used as an internal control used in a FTC-3000P PCR instrument (Funglyn). Using the 2^-ΔΔ^Ct method, target gene expression levels were normalized to the β-actin expression level before the relative levels of the target genes were compared.

### Flow cytometry cell apoptosis and cycle analysis

The CNE-2R and 6-10BR cells transfected with sh-FAM133B-2 or control siRNA were seeded into 6-well plates, harvested after 48h and rinsed with PBS twice. Cells were treated with 200μl binding buffer, 5μl Annexin V-FITC and 5μl propidium iodide (PI). After incubation in the dark for 30min at room temperature, the cell apoptotic rate was measured. The CNE-2R and 6-10BR cells transfected with CDK6 siRNA or control siRNA were seeded into 6-well plates, harvested after 48h and rinsed with PBS twice. Cells were treated with 200μl propidium iodide (PI). After incubation in the dark for 30min at room temperature, the cell apoptotic rate was measured by flow cytometry (Beckman) and analyzed by Flowjo Software. The experiments were performed independently three times, and a representative is shown.

The experiments were performed independently three times, and a representative is shown.

### Western blot protein analysis

Cells were lysed and heated at 95°C for 10min before electrophoresis/western blot analysis. The primary anti-CDK6 (14052-1-AP, Proteintech) antibodies and anti-GAPDH (60004-1-lg, Proteintech) antibodies were purchased from Proteintech and were recognized with anti-rabbit IgG peroxidase-conjugated antibody (10285-1-AP, Proteintech), followed by an enhanced chemiluminescence reaction (Thermo). Relative levels of proteins were quantified using densitometry with a Gel-Pro Analyzer (Media). The target bands over the GAPDH band were densitometrically quantified, as indicated under each band. All full-length unprocessed gels of immunoblots were provided in Supplementary Figure 2.

### Luciferase reporter assay

Luciferase reporters were generated based on the psiCHECK2 vector. To construct psiCHECK-FAM133B-2 or CDK6-WT or MUT, the part-length sequences of FAM133B-2 or CDK6-WT or MUT containing the putative miR-34a-5p binding site, were synthetized and cloned into the psiCHECK2 vector. The luciferase reporter was co-transfected with miR-34a-5p mimic, miR-34a-5p-MUT mimic, miR-34a-5p antagomiR, miR-34a-5p-MUT antagomiR or miR-NC into NPC cells by Lipofectamine 2000 according to the manufacturer’s guidelines. The relative luciferase activity was measured with the Dual-Luciferase Reporter Assay System (Promega) using Promega GloMax 20/20 luminometer. The relative luciferase activities were analyzed as reported previously.

### Statistical analysis

Quantitative RT-PCR, cell viability and luciferase reporter assays were performed in triplicate, the data are presented as the means, and the error bars indicate the S.D. Excel was used to process the data. Two way Anova was used to calculate statistical significance.

## Supplementary Material

Supplementary Figures
