# Supplementary material for: Long non-coding RNA FAM133B-2 represses the radio-resistance of nasopharyngeal cancer cells by targeting miR-34a-5p/CDK6 axis
Source: Aging (Albany NY). 2020 Sep 5;12(17):16936–50. doi: 10.18632/aging.103600 (PMC7521541; doi:10.18632/aging.103600)
Supplement: Supplementary Figures [file aging-12-103600-s001..pdf]

SUPPLEMENTARY FIGURES

geneID	geneLength	CNE-2-FPKM	CNE-2R-FPKM	log2 Ratio(CNE-2R/CNE-2)	Up-Down	P-value	FDR	gene name
NONHSAG001973.2	402	0.01	1.77	7.46760555	Up	2.80E-05	0.00015	lnc-WDR63-5
NONHSAG011558.2	546.22	0.01	1.56	7.285402219	Up	2.11E-07	1.37E-06	lnc-MSRB3-3
NONHSAG030149.2	1154.74	0.01	1.31	7.033423002	Up	5.50E-15	5.89E-14	lnc-CCDC150-1
NONHSAG033951.2	592	0.01	1.27	6.988684687	Up	8.53E-07	5.22E-06	lnc-APOBEC3G-1
NONHSAG037497.2	510.06	0.01	1.27	6.988684687	Up	1.39E-05	7.53E-05	lnc-DRD5-10
NONHSAG004953.2	463	0.01	1.23	6.942514505	Up	0.00011	0.00055	lnc-PGBD2-1
NONHSAG011555.2	731.15	0.01	1.2	6.906890596	Up	2.60E-08	1.81E-07	lnc-WIF1-6
NONHSAG038165.2	457	0.01	1.19	6.894817763	Up	0.00011	0.00055	lnc-ALB-7
NONHSAG050058.2	902.36	0.01	0.93	6.539158811	Up	2.60E-08	1.81E-07	lnc-ZMAT4-3
NONHSAG002120.2	592	0.01	0.89	6.475733431	Up	5.64E-05	0.00028	lnc-ARHGAP29-1
NONHSAG005214.2	754.32	0.09	7.47	6.375039431	Up	1.19E-47	3.55E-46	lnc-USP6NL-7
NONHSAG037523.2	1408.73	0.01	0.77	6.266786541	Up	2.40E-11	2.08E-10	lnc-CPEB2-18
NONHSAG032753.2	1491	0.01	0.77	6.266786541	Up	5.94E-12	5.37E-11	RUNX1-IT1
NONHSAG045008.2	3977	0.01	0.73	6.189824559	Up	6.24E-28	1.13E-26	lnc-GJE1-2
NONHSAG036567.2	2883.15	0.02	1.36	6.087462841	Up	3.11E-34	6.80E-33	LINC01322
NONHSAG002184.2	1151	0.01	0.64	6	Up	1.05E-07	6.96E-07	LINC01776
NONHSAG035579.2	1174.11	0.03	1.88	5.969626351	Up	4.22E-20	5.78E-19	LINC00973
NONHSAG005153.2	3370.89	0.34	2.57	2.918161708	Up	3.69E-47	1.09E-45	LINC00707
NONHSAG045007.2	1193.31	0.16	1.2	2.906890596	Up	3.56E-10	2.85E-09	lnc-GJE1-3
NONHSAG030940.2	1511	0.06	0.43	2.841302254	Up	0.00014	0.00067	lnc-PER2-4
NONHSAG013224.2	1980	0.11	0.78	2.8259706	Up	1.07E-09	8.28E-09	lnc-SOHLH2-1
NONHSAG044013.2	394.82	0.42	2.95	2.812253721	Up	1.35E-05	7.28E-05	lnc-FKBP1C-6
NONHSAG045053.2	4994	0.01	0.07	2.807354922	Up	0.00011	0.00055	HYMAI
NONHSAG041795.2	581.8	0.3	1.95	2.700439718	Up	6.11E-05	0.00031	lnc-ARHGAP26-4
NONHSAG048817.2	1013.77	2.59	16.75	2.693137093	Up	6.50E-65	2.72E-63	LINC00513
NONHSAG013698.2	844	0.28	1.75	2.64385619	Up	9.93E-08	6.61E-07	lnc-PCDH9-1
NONHSAG048207.2	2457	1.14	7.01	2.62038062	Up	2.18E-87	1.26E-85	lnc-FAM133B-2
NONHSAG036067.2	908	0.33	1.98	2.584962501	Up	4.80E-09	3.53E-08	lnc-PLXND1-3
NONHSAG007698.2	1776.06	0.07	0.41	2.550197083	Up	7.08E-07	4.38E-06	lnc-BTBD10-7
NONHSAG006784.2	2026.69	0.41	2.37	2.531191244	Up	3.33E-24	5.36E-23	SH3PXD2A-AS1

gene	lncRNA	lncRNA	mRNA	CNE-2R-FPKM_mRN	CNE-2-FPKM_mRN	LOG2(CNE-2R/CNE-2)
SPINK6	NONHSAG041876.2	NONHSAT104439.2	NM_001195290	3.01	34.57	-3.52
SPINK6	NONHSAG041876.2	NONHSAT104439.2	NM_205841	2.56	23.68	-3.21
SLPI	NONHSAG081984.1	NONHSAT189715.1	NM_003064	282.37	1314.53	-2.22
WISP2	NONHSAG031843.2	NONHSAT188799.1	NM_003881	6.74	50.35	-2.90
MXLOC_017622	LXLOC_017622	LTCONS_00036084	MTCONS_00036082	4.11	18.71	-2.19
NR4A1	LXLOC_017622	LTCONS_00036084	NM_173157	2.24	20.25	-3.18
SLC43A2	NONHSAG020465.2	NONHSAT175813.1	NM_152346	1.06	4.93	-2.22
MXLOC_015576	NONHSAG009098.2	NONHSAT160518.1	MTCONS_00032302	2.43	6.63	-1.45
RAB26	NONHSAG071429.1	NONHSAT172548.1	NM_014353	5.96	20.39	-1.77
CDYL2	NONHSAG020081.2	NONHSAT143903.2	NM_152342	1.51	7.56	-2.32
CD55	NONHSAG004121.2	NONHSAT150390.1	NM_000574	99.11	320.6	-1.69
CD55	NONHSAG004121.2	NONHSAT150390.1	NM_001114752	3.77	8.18	-1.12
C11orf86	NONHSAG062896.1	NONHSAT159286.1	NM_001136485	16.2	68.43	-2.08
CASP9	NONHSAG057532.1	NONHSAT151163.1	NM_001229	4.54	15.84	-1.80
MGST3	NONHSAG003338.2	NONHSAT150002.1	NM_004528	16.86	37.43	-1.15
KRT17	NONHSAG073663.1	NONHSAT176199.1	NM_000422	130.53	368.51	-1.50
VWA7	NONHSAG096054.1	NONHSAT211500.1	NM_025258	1.49	5.38	-1.85
NCOA3	NONHSAG081565.1	NONHSAT188832.1	NM_001174088	1.01	1.9	-0.91
NCOA3	NONHSAG081565.1	NONHSAT188832.1	NM_006534	2.8	4.79	-0.77
ADAMTSL4	NONHSAG002860.2	NONHSAT149788.1	NM_019032	2.48	11.62	-2.23
KRT17	NONHSAG021159.2	NONHSAT146577.2	NM_000422	130.53	368.51	-1.50
PANK3	NONHSAG042176.2	NONHSAT104991.2	NM_024594	25.96	64.19	-1.31
RAB11FIP3	NONHSAG018177.2	NONHSAT051779.2	NM_001142272	3.18	5.73	-0.85
RAB11FIP3	NONHSAG018177.2	NONHSAT051779.2	NM_014700	2	4.92	-1.30
PANK3	NONHSAG042176.2	NONHSAT104992.2	NM_024594	25.96	64.19	-1.31
MXLOC_041548	NONHSAG024566.2	NONHSAT180116.1	MTCONS_00084440	5.03	13.74	-1.45
ITGA2	NONHSAG040369.2	NONHSAT204144.1	NM_002203	13.36	6.27	1.09
VIM	NONHSAG005322.2	NONHSAT155167.1	NM_003380	257.69	58.36	2.14
MXLOC_091728	LXLOC_091728	LTCONS_00185489	MTCONS_00185490	4.99	1.61	1.63
ADI1	NONHSAG078385.1	NONHSAT184043.1	NM_018269	32.16	9.97	1.69
MXLOC_088284	LXLOC_088284	LTCONS_00178956	MTCONS_00178957	5.06	2.05	1.30
KRT81	NONHSAG011192.2	NONHSAT028360.2	NM_002281	14.69	3.08	2.25
ANTXR2	NONHSAG038301.2	NONHSAT097124.2	NM_058172	2.63	1.07	1.30
ANTXR2	NONHSAG038301.2	NONHSAT097124.2	NM_001145794	10.33	3.19	1.70
SLC4A7	NONHSAG034638.2	NONHSAT195513.1	NM_001258379	22.32	5.87	1.93
CDK6	NONHSAG048207.2	NONHSAT121988.2	NM_001145306	18.86	1.04	4.18

miRNA id	TPM(CNE-2)	TPM(CNE-2R)	P-value	FDR	CNE-2/CNE-2R
hsa-miR-29c-3p	273.93	7.17	1.65E-07	8.84E-07	38.20502092
hsa-miR-203a-3p	472.96	12.57	0.0506482	0.1119336	37.62609387
hsa-miR-6511a-3p	12.23	1.22	1.75E-30	1.67E-29	10.02459016
hsa-miR-34a-5p	52.035	5.42	2.29E-16	1.77E-15	9.600553506
hsa-miR-6888-3p	0.68	0.08	0.0085548	0.02227902	8.5
hsa-miR-190a-3p	9.11	2.38	2.96E-23	2.61E-22	3.827731092
hsa-miR-203a-5p	0.68	0.18	0.0085548	0.02230704	3.777777778
hsa-miR-4792	0.96	0.26	0.00218972	0.00620122	3.692307692
hsa-miR-576-3p	4.24	1.15	3.27E-11	2.20E-10	3.686956522
hsa-miR-193b-3p	4552.42	1238.45	0.438656	0.62929681	3.675901328
hsa-miR-3646	0.8	0.22	0.00540266	0.01445124	3.636363636
hsa-miR-210-5p	13.15	3.66	6.33E-31	6.07E-30	3.592896175
hsa-miR-6735-5p	0.92	0.26	0.00340782	0.00934446	3.538461538
hsa-miR-5196-3p	0.92	0.26	0.00340782	0.00933211	3.538461538
hsa-miR-6796-5p	0.44	0.13	0.0548188	0.11974644	3.384615385
hsa-miR-642a-5p	3.88	1.15	1.49E-09	9.21E-09	3.373913043
hsa-miR-346	37.81	11.23	6.76E-80	9.94E-79	3.366874443
hsa-miR-2116-5p	3.04	0.92	1.53E-07	8.32E-07	3.304347826
hsa-miR-6810-3p	1.88	0.57	4.05E-05	0.00016541	3.298245614
hsa-miR-629-5p	118.18	35.99	7.78E-239	1.90E-237	3.283689914
hsa-miR-30a-5p	786.29	74.54	0.438656	0.62973261	10.54856453
hsa-miR-200a-3p	71.82	22.07	2.21E-144	4.24E-143	3.25419121
hsa-miR-497-5p	72.74	22.51	5.40E-145	1.05E-143	3.231452688
novel_mir503	1.68	0.53	0.000144786	0.00052657	3.169811321
hsa-miR-3065-5p	50.6	16.25	5.92E-97	9.30E-96	3.113846154
hsa-miR-7641	0.28	0.09	0.1478852	0.28048126	3.111111111
hsa-miR-1225-5p	0.28	0.09	0.1478852	0.28022488	3.111111111
hsa-miR-5004-3p	0.4	0.13	0.0854916	0.17793583	3.076923077
hsa-miR-6813-5p	0.4	0.13	0.0854916	0.17775736	3.076923077
hsa-miR-4284	0.4	0.13	0.0854916	0.17757925	3.076923077
hsa-miR-1228-3p	2.96	0.97	7.41E-07	3.78E-06	3.051546392
hsa-miR-99a-5p	6983.19	2306.09	0	0	3.028151547
hsa-miR-99a-3p	11.07	3.66	9.15E-22	7.93E-21	3.024590164
hsa-miR-708-5p	0.12	0.04	0.438656	0.63148187	3
hsa-miR-5583-3p	0.12	0.04	0.438656	0.63104364	3

Supplementary Figure 1. The RNA-seq data of lnc-FAM133B-2, miR-34a-5p and CDK6

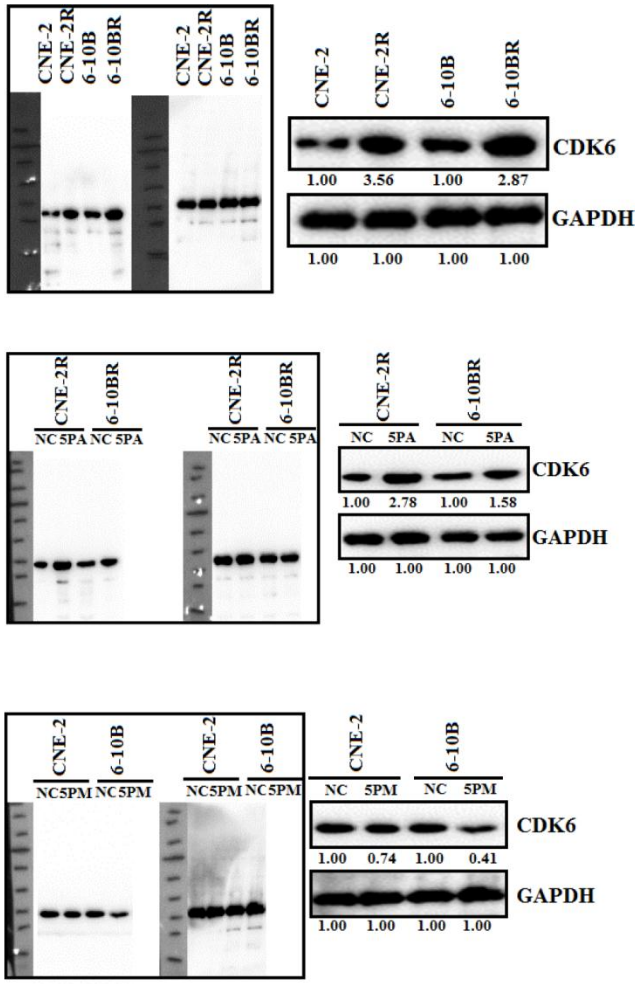

Supplementary Figure 2. The full-length gels of the Figure 4C western analyses used in the revised manuscript. The full-length gels of the Figure 4D western analyses used in the revised manuscript. The full-length gels of the Figure 4E western analyses used in the revised manuscript.

Gene	cell lines	qRT-PCR	RNA-seq
lnc-FAM133B-2	CNE-2	1.00±0.302	1.00
	CNE-2R	15.21±0.621	6.15
	6-10B	2.57±0.520	—
	6-10BR	7.25±0.911	—
miR-34a-5p	CNE-2	5.25±0.612	9.60
	CNE-2R	1.00±0.767	1.00
	6-10B	1.21±0.592	—
	6-10BR	0.54±1.211	—
CDK6	CNE-2	1.00±1.254	3.70
	CNE-2R	19.21±1.578	23.93
	6-10B	1.25±2.015	—
	6-10BR	7.55±2.205	—

Supplementary Figure 3. The expression data of lnc-FAM133B-2, miR-34a-5p and CDK6
